# Preparation of CSHW with Flue Gas Desulfurization Gypsum

**DOI:** 10.3390/ma15072691

**Published:** 2022-04-06

**Authors:** Xuemei Chen, Jianming Gao, Ye Wu, Qihong Wu, Li Luo

**Affiliations:** 1 School of Architecture and Civil Engineering, Chengdu University, Chengdu 610106, China; chenxuemei@cdu.edu.cn; 2 Jiangsu Key Laboratory of Construction Materials, School of Materials Science and Engineering, Southeast University, Nanjing 211189, China; 230179165@seu.edu.cn; 3 China MCC5 Group Corp. Ltd., Chengdu 610063, China; tangwenjie@cdu.edu.cn

**Keywords:** crystallography, manufacturing, growth

## Abstract

Calcium sulfate hemihydrate whiskers (CSHW), a multi-functional and high value-added building material, were prepared with flue gas desulfurization (FGD) gypsum by hydrothermal method, which could be a reasonable disposal of FGD gypsum. In order to obtain CSHW of a high aspect ratio, a series of manufacturing parameters such as reaction temperature, stirring speed, material–water ratio, and reaction time were investigated. The effect of stabilizing treatment and glycerol concentration on CSHW morphology were also studied by environmental scanning electron microscopy (ESEM) and statistical analysis. The results showed that the optimum preparing conditions of reaction temperature, stirring speed, water–material ratio, and reaction time were 160 °C, 200~300 rpm, 11:1 and 1 h, respectively. Furthermore, stabilizing treatment with octodecyl betaine was necessary for the preparation of CSHW. The final prepared whiskers had smooth surface, uniform morphology, a diameter of 260 nm, and a corresponding aspect ratio of 208.2. Moreover, the addition of glycerol reduced the activity of water, contributing to a lower reaction temperature and much smaller diameter.

## 1. Introduction

Calcium sulphate hemihydrate whiskers (CSHW) is a fibrous crystal formed by Ca^2+^ ions and SO_4_^2−^ ions. Since it is more likely to grow alternately along the C axis, the average aspect ratio of CSHW is usually more than 10 [1,2,3,4]. Due to its uniform structure, integral appearance, fine particles, high dispersion, high specific surface area and surface free energy, CSHW has desirable properties, such as high-temperature resistance, high chemical resistance, high strength and toughness. Additionally, CSHW has a strong market competitiveness as its price is only 1/200–1/300 of SiC, and it is easy for surface treatment. Therefore, CSHW have been widely used in papermaking, plastic, resin, rubber, friction materials, lightweight building materials and other fields [5,6]. The most important parameters of CSHW are the diameter and the aspect ratio, which have significant influence on the comprehensive performance of CSHW. Generally, CSHW with smaller diameter and higher aspect ratio can achieve excellent performance [7]. However, the diameter of the present CSHW can only achieve micrometer scale with a relatively low aspect ratio [8].

FDG is a by-product from the wet flue gas desulfurization process, which is widely adopted by sulfur-containing fuel (such as coal and residue oil) combustion systems. It was estimated that the annual production of FGD gypsum in China would reach 90 million tons by 2020 [9], ranking second among the most massive solid waste, just after fly ash. As FGD gypsum contains more than 93% CaSO_4_·2H_2_O, it can be used as a substitute of natural gypsum. Thereby, it has been used in cement as retarders [10,11,12], in land application [13] and building materials [14,15,16,17,18]. Presently, many researchers focus on the preparation of CSHW with the FGD gypsum. Hongjuan Sun [19] prepared CSHW of 3 to 22 μm width and 25 to 80 aspect ratio, by the atmospheric acidification method. The formation mechanism of the CSHW was that the two Ca–O–H bonds in the gypsum structure were broken and the H_2_O molecule released, and then bonded with the two S-formed novel Ca–O–S bonds, along the C axis of the H_2_O released [20]. Xiaoting Zhang [21] investigated the effect of the CuCl_2_ concentration on the CSHW crystal structure transition. Chengjun Liu [22] reported that both the average length and the aspect ratio of CSHW decreased in the presence of magnesium chloride, while a small quantity of citric acid or SDBS could improve the CSHW morphology. Although the theoretical research for the preparation of CSHW with FGD gypsum is very detailed, the influence of the preparation parameters for achieving CSHW of high aspect ratio in industrial production is rarely reported.

In this paper, FGD gypsum was used as the primary material to prepare CSHW by the hydrothermal method. A series of manufacturing parameters were investigated, aiming to achieve nano-scale CSHW. It was found that the addition of glycerol can further reduce the diameter of CSHW, and the stabilizing admixture was very important for morphology development. The average diameter and aspect ratio after treatment of octodecyl betaine were 260 nm and 208.2, respectively. The results of this paper could be used as guidelines for the actual production of CSHW with high aspect ratio. 

## 2. Experimental

### 2.1. Materials

**Figure 1:**
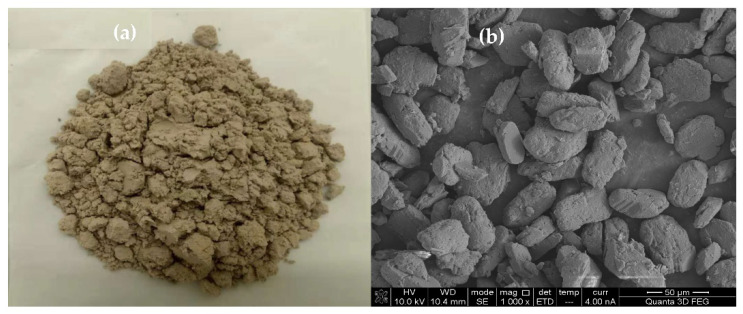
Apparent morphology (**a**), and SEM image (**b**), of FGD gypsum.

**Figure 2:**
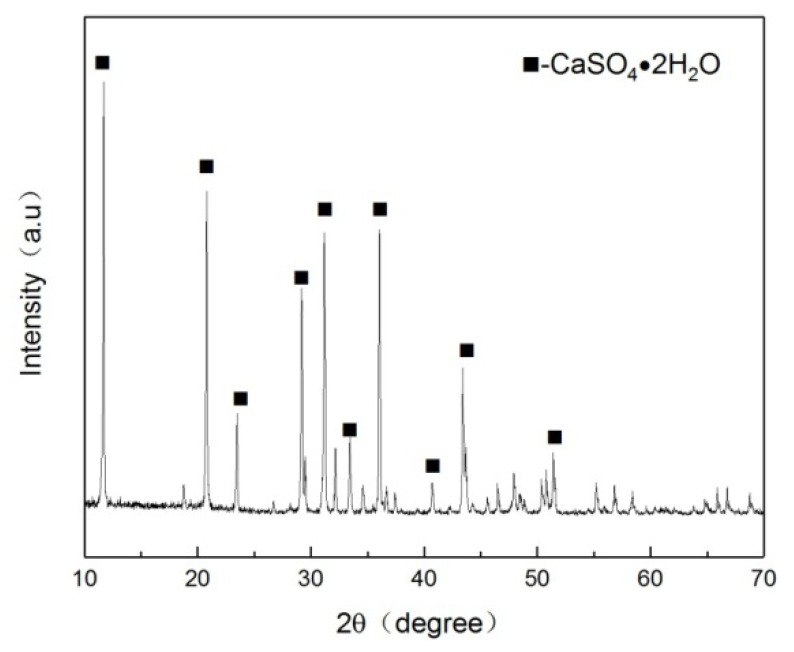
XRD patterns of FGD gypsum.

**Figure 3:**
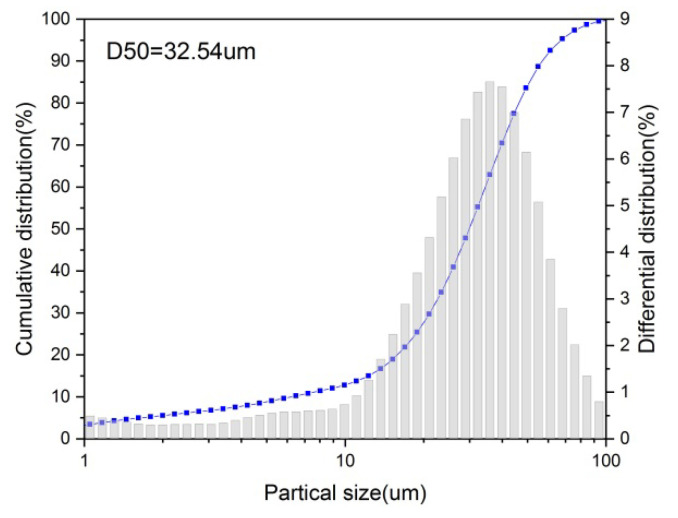
Particle size distribution curves of FGD gypsum.

FGD gypsum with yellow color (shown in Figure 1a) from the coal-fired power plant was used, which contained 13.5% free water. In addition, FGD gypsum contained 93.4% CaSO_4_·2H_2_O (see Figure 2), and its average particle size was 32.54 μm (see Figure 3). The chemical composition of the FGD gypsum is presented in Table 1. Analytical reagent of octodecyl betaine was used as a stabilizer and the analytical reagent of glycerol was used to modify the morphology of CSHW.

### 2.2. Methods

**Figure 4:**
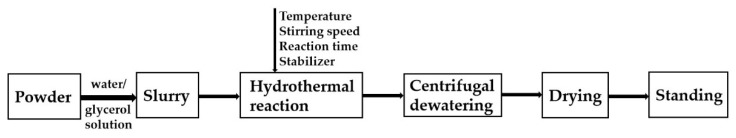
Experimental procedure of CSH whiskers preparation.

Gypsum slurry was firstly prepared according to the ratio of water to gypsum. After that, the mixture was poured into the autoclave, and was heated to the specific temperature when stirring at a certain speed. The stabilizer was added to the solution and stirred for a few minutes after reaction for a particular time at the setting temperature. Standing a little while with heat preservation, the slurry was taken out and immediately dehydrated by centrifugal machine. After drying at 150 °C to constant weight in the oven and then allowing it to stand for two days, the CSHW was finally prepared. The experimental procedure is shown in Figure 4, and the synthesis parameters are listed in Table 2.

The chemical composition of FGD gypsum was obtained by XRF (Axions, PANalytical B.V), and the size distribution of FGD gypsum was tested by Microtrac S3500 laser scattering technique. The morphology of prepared CSHW was examined by environmental scanning electron microscopy (Quanta 3D FEG, FEI). The average diameter, length and aspect ratio of CSHW were measured by the statistical method based on the ESEM observations.

## 3. The Growth Mechanism of CSHW Crystal 

There have been disputes over the formation mechanism of CSHW. The most conventional theory is dissolution–crystallization. According to the solubility curve, the solubility of CaSO_4_·2H_2_O is higher than that of CaSO_4_·0.5H_2_O under hydro-thermal conditions. The difference in the solubility is the driving force for the growth of CaSO_4_·0.5H_2_O crystal. Namely, the ions of Ca^2+^ and SO_4_^2−^ reach over-saturation for CaSO_4_·0.5H_2_O when dehydrate gypsum dissolves. Therefore, hemihydrate crystal nucleates and grows in the solution, and fibrous CSHW crystals are formed [23] if the crystal grows along the C axis after controlling the reaction condition.

As stated above and supported by the literature [24], the formation of crystals must experience three primary stages: the fluid medium achieving supersaturation, crystal nucleation and crystal growth. The crystal nucleation rate can be expressed as follows:

(1) $$I=B\cdot n\cdot \exp(-\Delta G{\left(i\right)}^{*}/kT)$$

(2) $$\Delta G{\left(i\right)}^{*}={\left(4\eta^{3}\gamma_{sf}^{3}\right)/\left[27k^{2}T^{2}\left(\ln c/c_{0}\right)\right]}^{2}$$

where ΔG(i)* is the energy needed for nucleation; *B* is the odds of crystal nucleus captured molecules in the solution; *n* is the number of solute molecules which are not united to form germ mass per unit volume in the growth system; *k* is Boltzmann constant; *T* is the absolute temperature of the system; *η* is the shape factor, whose value depends on the shape of the polyhedron; *γ_sf_* is the interfacial energy between crystals and solution; *c* and c_0_ are the saturation and supersaturation of the solution, respectively, under conditions of constant pressure and temperature.

The crystal growth process could be described as follows: solute diffuse to the crystal surface, adsorbed by the crystal surface, and finally stepping into the crystal lattice through reaction. The crystal growth rate is closely related to the diffusion of the solute and is proportional to the degree of supersaturation [25]. Therefore, the reaction condition of the hydro-thermal method including reaction temperature, stirring speed, material–water ratio and reaction time, in addition to the admixtures, has significant effect on the morphology of CSHW. 

## 4. Results and Discussion

### 4.1. The Effect of Reaction Temperature on the Morphology of CSHW 

**Figure 5:**
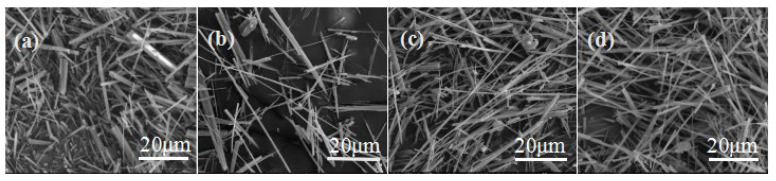
ESEM photos of CSHW at different reaction temperatures: (**a**) 120 °C; (**b**) 140 °C; (**c**) 160 °C; (**d**) 180 °C.

Gypsum can be divided into dihydrate, hemihydrate and anhydrite, and their solubility is closely related to temperature. For instance, the solubility of gypsum is, in order, CaSO_4_·0.5H_2_O > CaSO_4_·2H_2_O > CaSO_4_, when the temperature is below 100 °C. However, the solubility of gypsum is in order of CaSO_4_·2H_2_O > CaSO_4_·0.5H_2_O > CaSO_4_ [22,23] when the temperature is higher than 100 °C. From Section 3, it is clear that both the nucleation and growth of the whisker crystal are related to the temperature. Generally, high temperature is beneficial for the dissolution of gypsum, which will promote the crystal nucleation and growth to a certain extent. As shown in Figure 5, the CSHW with the lowest aspect ratio (9.75) and the largest diameter (2.58 μm) were observed at a temperature of 120 °C. As expected, the average aspect ratio increased and the average diameter decreased with the increase in temperature. The average aspect ratio came to 148.34 and the average diameter reached 560 nm when the temperature was 160 °C. Nevertheless, a decreasing trend in average aspect ratio (see Figure 5d) was noted, when the temperature was higher than 160 °C. Therefore, 160 °C was seemed to be the best reaction temperature.

### 4.2. The Effect of Stirring Speed on the Morphology of CSHW

**Figure 6:**
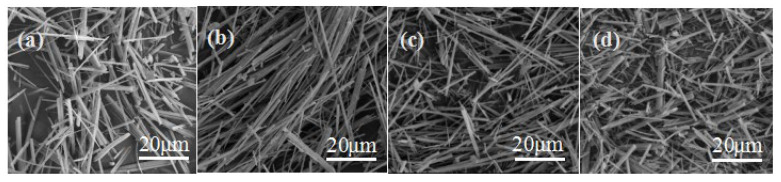
ESEM photos of CSHW at different stirring speeds: (**a**) 100 rpm, (**b**) 200 rpm, (**c**) 300 rpm, (**d**) 400 rpm.

Generally, mechanical disturbances are in favor of nucleation, but an increase in the intensity of agitation cannot always lead to an increase in nucleation [26,27]. The influence of stirring speed on morphology of CSHW is shown in Figure 6. The average aspect ratio of CSHW was 12.07, and its diameter was 1.96 μm when the stirring speed was 100 rpm. This may be explained by assuming that the stirring speed was too low, resulting in a lower nucleation rate. The aspect ratio increased with the increase of the stirring speed. It reached a maximum value of 146.05, and the average diameter was 390 nm, when the stirring speed was 200~300 rpm. However, the average aspect ratio decreased with a further increase in stirring speed, which was even lower than 10. Additionally, some whiskers were fractured and broken when the stirring speed was 400 rpm. It can be deduced that agitation effects can lead to the disruption of sub-nuclei or molecular clusters in the solution. High stirring speed was not conducive to the growth of CSHW. Therefore, 200~300 rpm is perhaps the optimum stirring speed.

### 4.3. The Effect of Water–Material Ratio on the Morphology of CSHW

**Figure 7:**
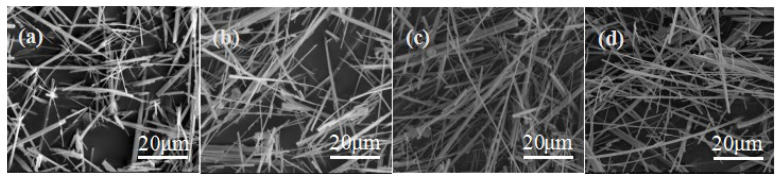
ESEM photos of CSHW in different water–material ratios: (**a**) 5:1, (**b**) 7:1, (**c**) 9:1, (**d**) 11:1.

The water–material ratio mentioned in the article is the mass ratio of water and FDG gypsum. The water–material ratio has a different influence on the morphology of CSHW. In one way, a lower water–material ratio is beneficial to improve the supersaturation of the solution, which would improve the nucleation and growth rate of whiskers. Conversely, the formation of a large amount of CSHW needs to absorb a quantity of heat, as the reaction is an endothermic process. If the temperature provided by the outside world is constant, the system temperature is bound to drop, and this phenomenon would be more significant at the lower water–material ratio. Consequently, the ion migration rate of the system decreased, resulting in a decline of the nucleation rate and the whisker growth rate. Additionally, the odds of secondary nucleation on the whisker surface increased, leading to large diameter due to excessive growth. As shown in Figure 7, the average aspect ratio of CSHW decreased and the average diameter increased gradually with the decrease in the water–material ratio. Obviously, the negative effect of a lower water–material ratio is predominant compared with its positive effect. Hence, the higher water–material ratio of 11:1 was chosen as the best water–material ratio.

### 4.4. The Effect of Reaction Time on Morphology of CSHW

**Figure 8:**
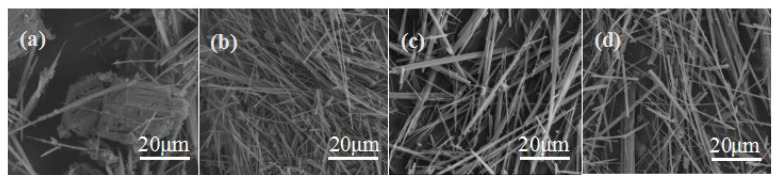
ESEM photos of CSHW at different reaction times: (**a**) 0.5 h, (**b**) 1 h, (**c**) 1.5 h, (**d**) 2 h.

It takes time to generate CSHW, and the needed reaction time will be different at different reaction temperatures. If the reaction time is too short, it can cause an inadequate reaction, but if it is too long, secondary crystallization of whisker is more likely to occur, leading to a lower aspect ratio. As shown in Figure 8, the whiskers were not completely generated when the reaction time was 0.5 h at the reaction temperature of 160 °C. The average aspect ratio of CSHW increased gradually with the increase in reaction time. The average aspect ratio of CSHW increased to 127.4, and the average diameter decreased to 0.42 μm, when the reaction time was 1 h. However, the average aspect ratio decreased and the average diameter increased with a further increase in the reaction time. Therefore, 1 h would be the optimal reaction time. 

### 4.5. XRD Patterns under Different Preparation Conditions

**Figure 9:**
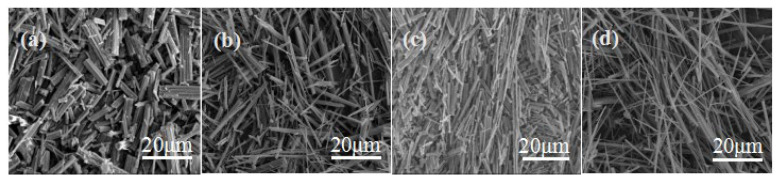
ESEM photos of CSHW with different aspect ratios. (The preparing parameters of (**a**–**d**) were shown in Table 3).

**Figure 10:**
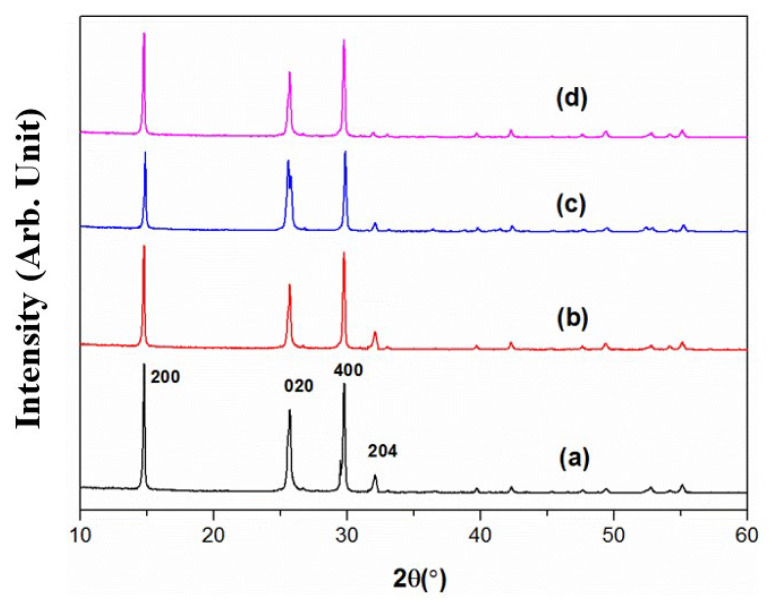
XRD patterns of CSHW with different aspect ratios. (The preparing parameters of (**a**–**d**) were shown in Table 3).

CSHW with different aspect ratios were obtained by altering the preparing parameters. The morphology of CSHW with different aspect ratios are shown in Figure 9, and the corresponding preparing parameters are depicted in Table 3. All of the synthesized CSHW showed similar patterns, as shown in Figure 10. All spectra were indexed to phase of hemihydrate (CaSO_4_·0.5H_2_O, PDF-#14-0453). No characteristic peaks relating to the gypsum or impurities were detected, indicating complete conversion of gypsum to hemihydrate. Nevertheless, it is worth noting that the intensity of the (204) crystal plane in relation to that of the (200) crystal plane decreased gradually with an increase in average aspect ratio. It is reported that the intensity of the XRD spectrum diffraction peak is related to the morphology of CSHW [28]. The results confirm the preferential growth of the CSHW crystal along the C axis.

### 4.6. Stabilization of CSHW

**Figure 11:**
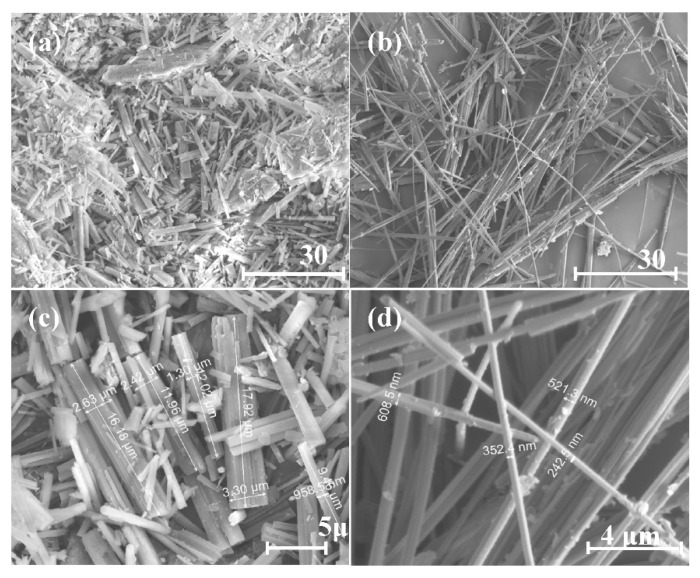
ESEM photos of samples before and after stabilizing: (**a**,**c**) before stabilizing; (**b**,**d**) after stabilizing. Figure 11c,d are the enlarged pictures of Figure 11a,b, respectively.

**Figure 12:**
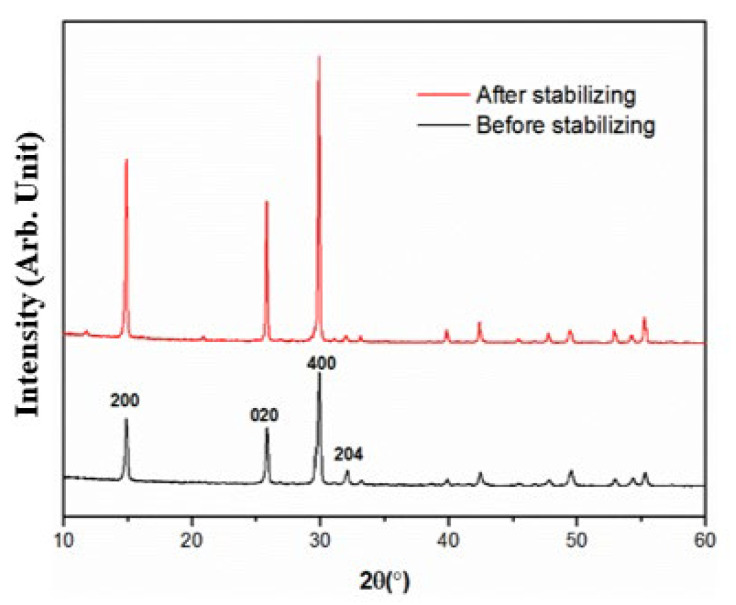
XRD patterns of the samples before and after stabilizing.

The optimum preparing parameters for CSHW were reaction temperature 160 °C, stirring speed 200~300 rpm, water–material ratio 11:1 and reaction time 1 h. As hemihydrate whisker tends to hydrate into gypsum in aqueous solution or moist air due to the inner porosity and the Ca^2+^ located at the surface active site, stabilizing treatment is necessary. Organic acids including monic acid, dibasic acid, and ternary acid were used to stabilize the prepared CSHW. It was found that octodecyl betaine had a significant effect on stabilizing CSHW. The carboxyl group (–COOH) in octodecyl betaine is likely to ionize (–COO–) and react with Ca^2+^ forming (–COO)_2_Ca, which hinders the hydration of whisker. Figure 11 shows the ESEM photographs of samples before and after stabilizing treatment. As can be seen from the photographs, the CSHW without stabilizing treatment hydrated seriously, with uneven morphology, coarse diameter and shorter length, while the CSHW after stabilizing treatment by octodecyl betaine had a completely uniform morphology. The average aspect ratio was as high as 208.2, and the average diameter was 0.26 μm. The size of whiskers before and after stabilizing treatment are shown in Table 4, and the XRD spectra is presented in Figure 12. It can be seen that the intensity of the (204) crystal plane in relation to that of the (200) crystal plane in the XRD pattern of CSHW without stabilizing treatment was remarkably higher than that of CSHW after stabilizing treatment. This suggests that CSHW, after stabilizing treatment, have a higher aspect ratio, which is consistent with ESEM photographs. 

### 4.7. Effect of Glycerol Concentration on the Morphology of Whiskers

The equilibrium equation of gypsum solution can be described as: $CaSO_{4}\cdot nH_{2}O\Leftrightarrow Ca^{2+}+SO_{4}{}^{2-}+nH_{2}O$. The solubility product constant can be expressed as: $K_{sp}=(a_{Ca^{2+}})(a_{SO_{4}^{2-}{}_{}}){\left(a_{H_{2}O}\right)}^{n}$, where *a* is activity, and *n* is equal to 0, 0.5, and 2, which represent anhydrite, hemihydrate and dihydrate, respectively [29]. The reaction of dihydrate to hemihydrate in supersaturated solution at high temperature is expressed as:

(3) $$CaSO_{4}\cdot 2H_{2}O(s)=CaSO_{4}\cdot 0.5H_{2}O(s)+1.5H_{2}O(l).\;\;K_{CaSO_{4}\cdot 2H_{2}O\Rightarrow CaSO_{4}\cdot 0.5H_{2}O}=K_{sp,CaSO_{4}\cdot 2H_{2}O}/K_{sp,CaSO_{4}\cdot 0.5H_{2}O}={\left(a_{H_{2}O}\right)}^{1.5}$$

**Figure 13:**
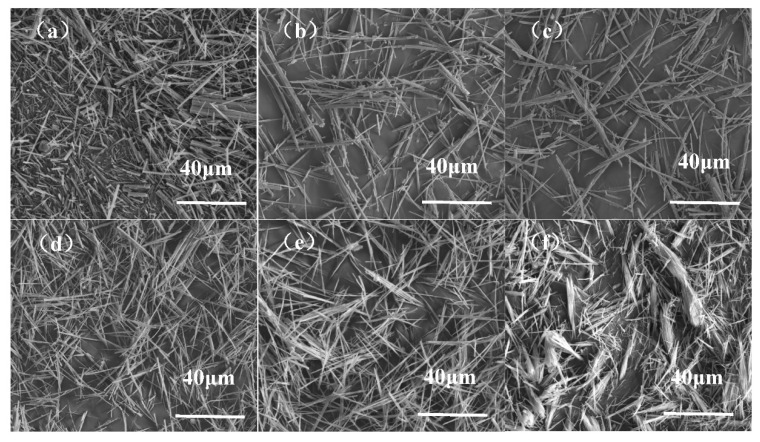
SEM images of samples prepared in different glycerol concentrations: (**a**) 0%, (**b**) 20%, (**c**) 40%, (**d**) 60%, (**e**) 80%, (**f**) 90%.

Therefore, the transformation of dihydrate to hemihydrate depends on the activity of water. The water activity of a solution is defined as the ratio of the vapor pressure of water over the solution to that over the pure water at the same temperature [30]: 

$\alpha_{w}=p_{H_{2}O}/P_{H_{2}O}^{0}$. As the vapor pressure of pure water at certain temperature is fixed, the water activity is proportional to the vapor pressure of water over the solution. In theory, the vapor pressure of water over the solution is related to the water contents. Thus, it makes sense that an increase in glycerol concentration would lead to a reduction in water activity. The hydroxide radical in glycerol would combine calcium sulfate dihydrate to form a hydrogen bond, which is in favor of gypsum dissolution, resulting in higher supersaturation. The supersaturation is the driving force for the precipitation of calcium sulfate hemihydrate. Therefore, the CSHW grows fast in glycerol solution due to higher supersaturation. At the same time, some anions of glycerol are selectively chemisorbed on the surface of crystals by complexing with Ca^2+^, which enables hemihydrate to grow along the C axis and eventually form fibrous crystals. As shown in Figure 13a–f, the average diameter of whiskers decreased gradually and the aspect ratio increased as an increase in glycerol concentration. The specified size of generated whiskers was illustrated in Table 5. The higher the glycerol concentration, the higher the average aspect ratio. However, the generated whiskers were broom-like when the glycerol concentration was 90%. This was probably caused by a large number of whiskers reunited because of its high surface area and surface activation energy. Therefore, an increase in the glycerol concentration is the most effective way to obtain CSHW of higher aspect ratio during the synthesis process. Taking the costs into account, 60% could be the optimal glycerol concentration.

**Figure 14:**
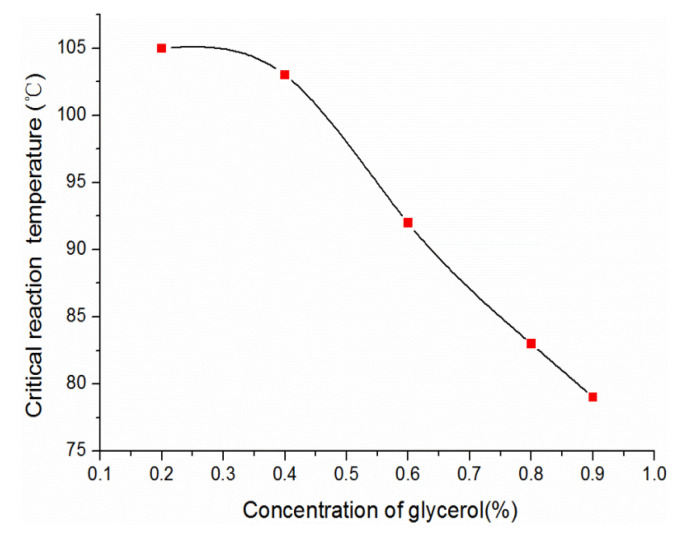
The critical reaction temperature in different glycerol concentrations.

The glycerol concentration has a significant effect on the critical reaction temperature. Keeping the preparing parameters unchanged, the formation temperature of CSHW was measured by altering the glycerol concentration, and the results are shown in Figure 14. It was apparent that the critical temperature gradually reduced with the increase in glycerol concentration, which confirmed that glycerol had a negative effect on the activity of water in the solution [27]. Because *K_sp_* is only a function of temperature, water activity would be the only determinant of the phase-transition temperature indicated by Equation (3). The phase transition can proceed spontaneously when α_w_ is reduced to a required level, and α_w_ depends on the composition of the electrolyte solution. Hence, the phase-transition temperature of each phase varies with the change of electrolyte concentrations.

**Figure 15:**
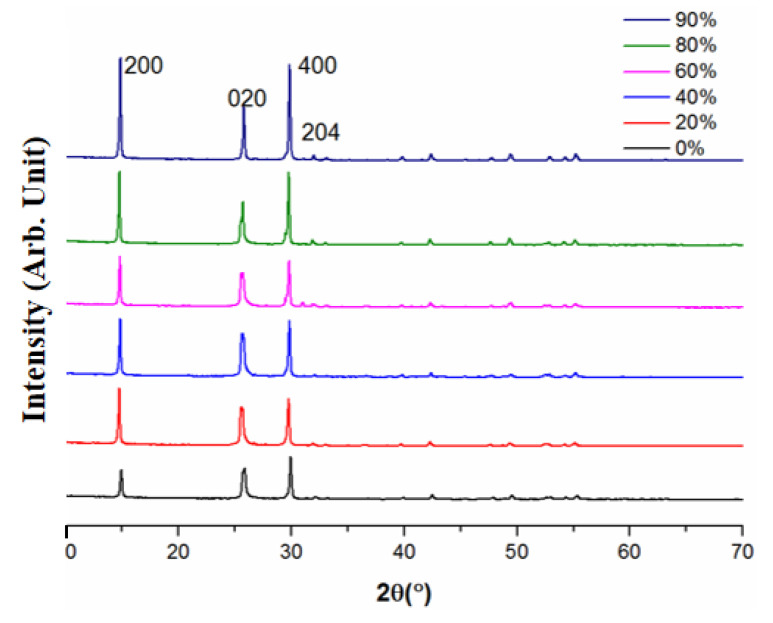
The XRD patterns of samples prepared in different glycerol concentrations.

The XRD patterns of samples prepared with different glycerol concentrations are depicted in Figure 15. Similar patterns were observed in all samples, and all spectra were indexed to CaSO_4_·0.5H_2_O as displayed in Figure 8. With the increase in glycerol concentration in Figure 15, the intensity of the (400) and (020) crystal planes were kept almost unchanged, while the intensity of the (200) and (204) crystal planes decreased gradually. The results reconfirmed the preferential growth of the crystal along the C axis.

## 5. Conclusions

In this study, the CSHW was prepared with flue gas desulfurization gypsum by the hydro-thermal method. A series of preparing parameters were investigated and the influence of stabilizing treatment and glycerol were also discussed, which can be used as guidelines for factory production. The results and discussion lead to the following conclusions:

(1) CSHW were prepared by hydro-thermal method using FGD gypsum as the primary material. The optimum preparing parameters were reaction temperature 160 °C, stirring speed 200~300 rpm, water–material ratio 11:1 and reaction time 1 h;
(2) Octodecyl betaine had a significant effect on stabilizing CSHW. According to the statistical analysis, CSHW after stabilizing treatment by octodecyl betaine had a complete uniform morphology. Its average diameter was 0.26 μm and the average aspect ratio was as high as 208.2;
(3) The diameter of whiskers prepared in glycerol solution reduced even lower than 0.1 μm, reaching nanometer scale. An increase in glycerol concentration led to a reduction in critical reaction temperature, in addition to the average diameter and the aspect ratio. The addition of glycerol may be the most effective way to obtain whisker of higher aspect ratio since it can significantly affect the activity of water;
(4) The intensity of the XRD spectrum diffraction peak was related to the morphology of CSHW. With the increase in aspect ratio, the intensity of the (204) crystal plane in relation to that of the (200) crystal plane decreased gradually, confirming the preferential growth of the crystal along the C axis.

## Figures and Tables

**Table 1:** Chemical composition (by mass %) of FGD gypsum.

Composition (%)	SO_3_	CaO	MgO	Fe_2_O_3_	Al_2_O_3_	N_2_O	K_2_O	H_2_O	Others
FGD	44.36	31.36	0.56	0.18	0.36	0.05	0.13	18.65	2.53

**Table 2:** Synthesis parameters for samples.

Sample	A1-4	B1-4	C1-4	D1-4
Reaction temperature/°C	120/140/160/180	140	140	140
Stirring speed/rpm	200	100/200/300/400	100	100
Reaction time/h	2.5	2.5	0.5/1/1.5/2.0	2.5
Water–material ratio	7:1	7:1	7:1	5/7/9/11:1

**Table 3:** The preparing parameters of CSHW.

Preparing Parameter	a	b	c	d
Temperature/°C	120	140	180	160
Stirring speed/rpm	400	100	400	200
Reaction time/h	2.5	2.5	1.5	1
Water–material ratio	11:1	7:1	5:1	11:1
Average aspect ratio	5.3	15.6	22.4	195.6
Average diameter/um	3.7	1.8	1.2	0.36

**Table 4:** The size of the CSHW before and after stabilizing.

Stabilizing Treatment	Average Length (μm)	Average Diameter (μm)	Average Aspect Ratio
Before stabilizing	14.68	2.46	6.02
After stabilizing	96.57	0.26	208.2

**Table 5:** The size of generated whiskers in different glycerol concentrations.

Glycerol wt%	0%	20%	40%	60%	80%	90%
Average length (μm)	49.3~60.8	47.0~62.1	58.3~74.7	52.6~56.0	43.9~46.2	26.8~32.0
Average diameter (nm)	1200~1560	789~850	589~653	186~260	146~201	94~120
Average aspect ratio	31.6~50.6	55.3~78.7	89.3~126.8	202.3~301.0	218.5~316.4	223.3~340.4

Note: the other synthesis parameters were 120 °C, 2.5 h, 11:1 and 200 rmp.

## Data Availability

Data sharing not applicable.
